# Ens-PPI: A Novel Ensemble Classifier for Predicting the Interactions of Proteins Using Autocovariance Transformation from PSSM

**DOI:** 10.1155/2016/4563524

**Published:** 2016-06-29

**Authors:** Zhen-Guo Gao, Lei Wang, Shi-Xiong Xia, Zhu-Hong You, Xin Yan, Yong Zhou

**Affiliations:** ^1^School of Computer Science and Technology, China University of Mining and Technology, Xuzhou, Jiangsu 221116, China; ^2^College of Information Science and Engineering, Zaozhuang University, Zaozhuang, Shandong 277100, China; ^3^School of Foreign Languages, Zaozhuang University, Zaozhuang, Shandong 277100, China

## Abstract

Protein-Protein Interactions (PPIs) play vital roles in most biological activities. Although the development of high-throughput biological technologies has generated considerable PPI data for various organisms, many problems are still far from being solved. A number of computational methods based on machine learning have been developed to facilitate the identification of novel PPIs. In this study, a novel predictor was designed using the Rotation Forest (RF) algorithm combined with Autocovariance (AC) features extracted from the Position-Specific Scoring Matrix (PSSM). More specifically, the PSSMs are generated using the information of protein amino acids sequence. Then, an effective sequence-based features representation, Autocovariance, is employed to extract features from PSSMs. Finally, the RF model is used as a classifier to distinguish between the interacting and noninteracting protein pairs. The proposed method achieves promising prediction performance when performed on the PPIs of* Yeast*,* H*.* pylori*, and* independent datasets*. The good results show that the proposed model is suitable for PPIs prediction and could also provide a useful supplementary tool for solving other bioinformatics problems.

## 1. Introduction

Proteins are the most versatile and important macromolecules in life. They are vital for nearly all of the activity in the cell, including signaling cascades, metabolic cycles, and DNA transcription and replication [1]. Researchers found out that proteins rarely act as isolated agents to achieve their function. As expected, proteins are mutually matched with each other, forming a huge and complex network of Protein-Protein Interactions (PPIs) [2]. Therefore, research on PPIs has become the core issue of systems biology [3, 4].

So far, a variety of experimental techniques have been developed and designed for the detection of PPIs. The high-throughput techniques including Yeast Two-Hybrid (Y2H) screen [5–7], Tandem Affinity Purification (TAP) [2], and Mass Spectrometric Protein Complex Identification (MS-PCI) [6] spend considerable amounts of time, money, and manpower for detecting PPIs. In addition, PPIs obtained by biological experiments at present can only cover a small part of the whole PPIs network [8]. Therefore, the development of reliable computational methods which can improve the recognition efficiency has important significance [9–11].

A large number of* in silico* methods for predicting PPI have emerged [12–14]. These methods are usually based on the information of gene neighboring [15], gene coexpression [15], phylogenetic relationship [16], gene fusion events [17], three-dimensional structural information [18], and so on [19]. However, the application of these methods is limited [20, 21], because they need to rely on preknowledge of the protein. Recently, the methods based on the sequence information of protein amino acids for detecting PPI have been proposed [22–24]. For example, You et al. [25] used only protein sequence information to predict PPI, in which a kind of method called PCA-EELM (Principal Component Analysis-Ensemble Extreme Learning Machine) is designed. When performed on the PPIs data of* Saccharomyces cerevisiae*, this model yields 87.00% prediction accuracy, 86.15% sensitivity, and 87.59% precision. Martin et al. [26] designed a model to detect PPIs by using the extended signature descriptor, which was extended to protein pairs. In order to verify the predictive ability of this method, when using 10-fold cross-validation applied on the* H*.* pylori* and* Yeast* datasets, the accuracy of this method is from 70% to 80%. Shen et al. [11] considered the residues local environments and designed the conjoint triad method. When performed on* human* PPIs dataset, this method has yielded 83.9% accuracy. Guo et al. [9] combined Support Vector Machine classifier with Automatic Covariance features extracted from the protein sequences to predict PPIs in* Saccharomyces cerevisiae*. The average prediction accuracy of the method reached 86.55%.

In this study, we presented a sequence-based method which combines the RF classifier and Autocovariance (AC) algorithm to predict the interacting protein pairs [9, 27, 28]. A novel protein feature representation is derived from Position-Specific Scoring Matrix (PSSM) [29], which gives the log-odds score of specific residue replacement based on specific location of evolutionary information. Then, an effective sequence-based protein representation, Autocovariance, is employed to extract features from PSSMs. The interaction among a certain number of amino acid sequences was calculated by AC algorithm. Thus, this model took into account the proximity effect and made it possible to find patterns throughout the sequence. Finally, the ensemble RF classifier is established, which is using the PSSM-derived features as input. In the experiments, the proposed model was evaluated on* Yeast* and* H*.* pylori* PPI datasets. The experiment results show that our model achieved 97.77% and 84.84% prediction accuracy with 95.57% and 82.77% sensitivity on these two datasets. In addition, we evaluate the proposed model on independent datasets of the* C*.* elegans*,* E*.* coli*,* H*.* sapiens*, and* M*.* musculus* PPIs and achieved 96.01%, 97.73%, 98.30%, and 96.81% prediction accuracy, respectively.

## 2. Materials and Methodology

### 2.1. Data Sources

In the experiments, we used nonredundant* Yeast* data, which was gathered in* Saccharomyces cerevisiae* core subset of the Database of Interacting Proteins (DIP) [30], and the version is DIP* 20070219* by Guo et al. [9]. Two methods, Paralogous Verification Method (PVM) and Expression Profile Reliability (EPR) [31], have proven the reliability of the core subset. There are 5966 interaction pairs contained in the core subset. Sequences with less than 50 amino acid residues were removed because they might just be fragments. The final positive dataset was comprised of the remaining 5943 protein pairs. The CD-Hit [32, 33] algorithm was further used with less than forty percent identity to decrease pairwise sequence redundancy. By doing this, the rest of the 5594 protein pairs constructed the positive dataset. We chose 5594 additional protein pairs in different subcellular localization to construct the negative dataset. Finally, the complete dataset was constructed; it was composed of 11188 protein pairs, half of which were positive and the other half were negative.

We also tested our method using two-hybrid measurements of* H*.* pylori* introduced by Rain et al. [34]. The* H*.* pylori* dataset (available at http://www.cs.sandia.gov/~smartin/software.html) contains 2916 protein pairs. There are interacting pairs and noninteracting pairs, each accounting for fifty percent. This dataset provides a platform for comparing our approach and other approaches [25, 26, 35–38].

### 2.2. Position-Specific Scoring Matrix (PSSM)

Position-Specific Scoring Matrix is first used in the detection of distantly related protein, which is proposed by Gribskov et al. [29]. Its feasibility has been verified in protein secondary structure prediction [39], prediction of disordered regions [40], and protein binding site prediction [41]. Structure of a PSSM is *L* rows and 20 columns. Suppose that PSSM = {*θ*_*i*,*j*_ : *i* = 1,…, *L*, *j* = 1,…, 20}. Rows of the matrix represent the protein residues and columns represent the naive amino acids. Each matrix can be represented by the following formula:

(1) $$\begin{array}{c} \text{PSSM}=\left[\begin{array}{cccc} \theta_{1,1} & \theta_{1,2} & \cdots & \theta_{1,20}\\ \theta_{2,1} & \theta_{2,2} & \cdots & \theta_{2,20}\\ \vdots & \vdots & \vdots & \vdots \\ \theta_{L,1} & \theta_{L,2} & \cdots & \theta_{L,20} \end{array}\right], \end{array}$$

where *L* is the length of the corresponding protein sequence and *θ*_*i*,*j*_ in the *i* row of PSSM meant the probability of the *i*th residue being mutated into type *j* of 20 native amino acids during the procession of evolutionary information in the protein from multiple sequence alignments.

In this experiment, we introduced the Position-Specific Iterated BLAST (PSI-BLAST) program [42] and* SwissProt* dataset on a local machine to produce PSSMs. PSI-BLAST is more sensitive compared to BLAST, particularly in the discovery of new members of a protein family. To generate the PSSM, PSI-BLAST needs sequence contrast with very high sensitivity between the input proteins and the proteins in the database, and all sequence entries in the* SwissProt* database have been carefully verified by computer tools and access to relevant literature through the experience of molecular biologists and protein chemists, so we put* SwissProt* database as the optimal comparison database in the experiment. And to get broad and high homologous sequences, we held the other parameters constant, where the *e*-value is set to 0.001 and the number of iterations is set to 3, respectively. Applications of PSI-BLAST and* SwissProt* database can be downloaded from http://blast.ncbi.nlm.nih.gov/Blast.cgi.

### 2.3. Autocovariance (AC)

As one of the most effective analyzing sequences of vectors statistical tools, the AC has been widely used in protein family classification by researchers [43, 44], prediction of secondary structure content [45, 46], and protein interaction prediction [9]. AC is a variable expressed in a given protein sequence of two residues' average correlation, which can be calculated by

(2) AC⁡λ,lg=1L−lg∑λ=1L−lgMλ,θ−1L∑λ=1LMλ,θ·Mλ+lg,θ−1L∑λ=1LMλ,θ,

where *lg* is the distance between residues, *λ* represents the *λ*th amino acid, *L* denotes the length of the protein sequence, and *M*_*λ*,*θ*_ indicates the matrix score of amino acid *λ* at position *θ*.

Using the above expression, the value of AC variable *M* can be figured out: *M* = *lg* × *N*, where *N* is the number of descriptors. When all the data in the database complete the operation, each protein sequence was represented as a vector of AC variables; a protein pair was characterized by concatenating the vectors of two proteins in this protein pair.

### 2.4. Rotation Forest Classifier

Rotation Forest (RF) is a popular ensemble classifier and this idea originated from Random Forests classifier. Each decision tree in Rotation Forest is trained on the dataset in a rotated feature space. As a decision tree learning algorithm establishes the classification regions using hyperplanes parallel to the feature axes and a small rotation of axes may build an entirely different tree, the diversity of RF can be guaranteed by the transformation. Thus, RF model can enhance the accuracy for individual classifier and the diversity in the ensemble at the same time. It is more robust compared to the previously proposed ensemble systems, such as Random Forest [32, 47], Bagging [33, 48], and Boosting [49]. The RF algorithm is described as follows.

Assuming {*x*_*i*_, *y*_*i*_} contains *N* training samples, wherein *x*_*i*_ = (*x*_*i*1_, *x*_*i*2_,…, *x*_*iD*_) is a *D*-dimensional feature vector. Suppose that *X* is the training sample set (*n* × *D* matrix), which is composed of *n* observation feature vector composition; *S* denote the feature set, and *Y* denote the corresponding labels, and then *X* = (*x*_1_, *x*_2_,…,*x*_*n*_)^*T*^, *Y* = (*y*_1_, *y*_2_,…,*y*_*n*_)^*T*^. Assume a feature set with an appropriate factor randomly divided into *K* subsets of the same size; in this case, the decision trees *L* in the forest can be expressed as *T*_1_, *T*_2_,…, *T*_*L*_, respectively. The execution steps of the training set for a single classifier *T*_*i*_ are shown below:

(1) Select the appropriate parameter *K* which is a factor of *n*; let *S* be randomly divided into *K* parts of the disjoint subsets; each subset contains a number of features, *C* = *n*/*k*.
(2) From the training dataset *X*, select the corresponding column of the feature in the subset *T*_*i*,*j*_ and form a new matrix *X*_*i*,*j*_, followed by a bootstrap subset of objects extracting 75 percent of *X* constituting a new training set *X*_*i*,*j*_′.
(3) Matrix *X*_*i*,*j*_′ is used as the feature transform for producing the coefficients in a matrix *M*_*i*,*j*_, with *j*th column coefficient as the characteristic *j*th component.
(4) The coefficients obtained in the matrix *M*_*i*,*j*_ are constructed as a sparse rotation matrix *R*_*i*_, which is expressed as follows:
  (3) Ri=λi,11,…,λi,1C10⋯00λi,21,…,λi,2C2⋯0⋮⋮⋱⋮00⋯λi,k1,…,λi,kCk.

In the prediction period, the test sample *x*, generated by the classifier *T*_*i*_ of *d*_*i*,*j*_(*XR*_*i*_^*λ*^) to determine *x*, belongs to class *y*_*i*_. Next, the class of confidence is calculated by means of the average combination, and the formula is as follows:

(4) $$\begin{array}{c} \mu_{j}\left(\;x\;\right)=\frac{1}{L}\overset{L}{\underset{i=1}{\sum }}d_{i,j}\left(\;XR_{i}^{\lambda }\;\right). \end{array}$$

Then, assign the category with the largest *μ*_*j*_(*x*) value to *x*.

## 3. Results and Discussions

### 3.1. Evaluation Measures

**Figure 1:**
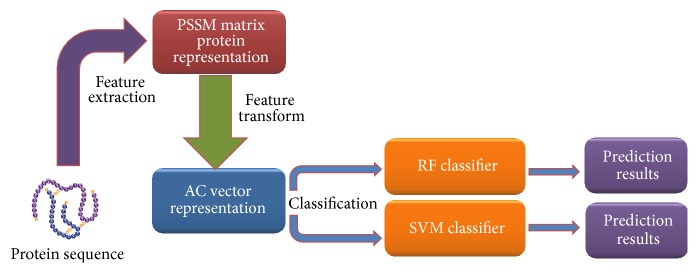
The workflow of our method.

In this section, 5-fold cross-validation is used to evaluate the performance of the proposed method, in which all samples are split into five subsets. Therefore, one subset is the test set and the remaining four subsets are the training set. Evaluation criteria used in our study include overall prediction accuracy (Accu.), sensitivity (Sen.), precision (Prec.), and Matthews correlation coefficient (MCC). The calculation formulas are listed below:

(5) Accu.=TP+TNTP+TN+FP+FNSen.=TPTP+FNPrec.=TPTP+FPMCC=TP×TN−FP×FNTP+FPTP+FNTN+FPTN+FN,

where True Positive (TP) represents the number of samples that are correctly detected as positive, True Negative (TN) represents the number of samples that are correctly detected as negative, False Positive (FP) represents the number of samples that are incorrectly detected as positive, and False Negative (FN) represents the number of samples that are incorrectly detected as negative. We also produce Receiver Operating Characteristic (ROC) [50] curves to assess the capability of the classifier. Typically, the threshold value of the classifier is 0.5 by default. When a new set of prediction results is accepted, the threshold value will be changed with the True Positive Rate versus the False Positive Rate; this change can be drawn out with graphics. In addition, the Area Under a Curve (AUC), with score ranges from 0 to 1, can also be expressed by the ROC curve. When a predictor of the AUC value is greater than another predictor, this predictor is regarded as a better one. The workflow of our method is shown in Figure 1.

### 3.2. Assessment of Prediction Ability

**Figure 2:**
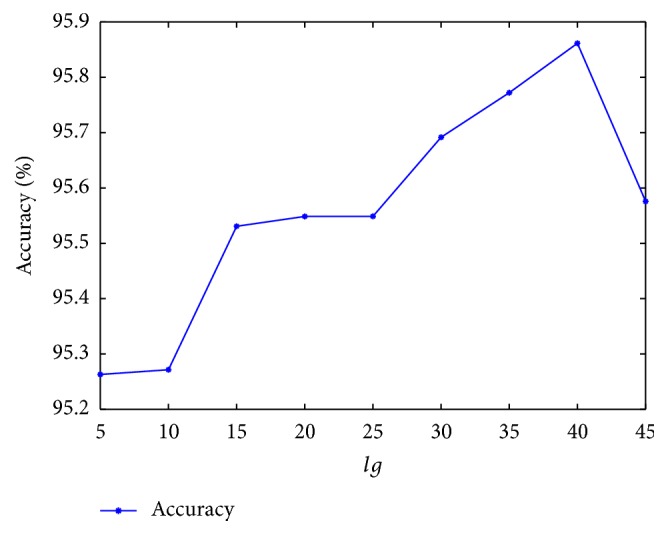
The average prediction accuracy corresponding to different *lg* of the AC algorithm in the proposed model.

In order to achieve better results in the experiment, we used the grid search method to explore the parameters of the proposed model; concrete has parameter *lg* value for AC and parameters *K* and *L* value for RF. Firstly, we discuss the parameters of AC; the maximal possible *lg* is the shortest sequence length (50 amino acids) on the* Yeast* dataset. In this experiment, several *lg*s (*lg* = 5,10,15,20,25,30,35,40,45) were evaluated in order to achieve the best performance of the protein sequences. The prediction results were shown in Figure 2. As seen from the curve in the graph, the prediction accuracy gradually increases when the parameters *lg* of the AC algorithm change from 5 to 40, and it decreases when the *lg* value changes from 40 to 45. There is a peak point with an average accuracy of 95.86% when the value of *lg* was 40. We can draw a conclusion; when the parameters *lg* of the AC algorithm are less than 40 or the number of amino acids is less than 40, protein sequences will lose some useful information, but larger *lg* may introduce noise rather than improvnig the performance of the model. So we set the value of *lg* as 40.

**Figure 3:**
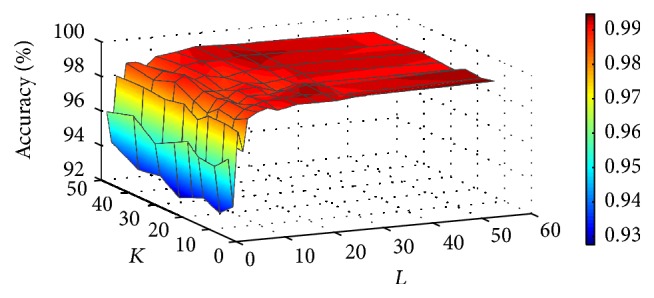
Accuracy surface obtained from Rotation Forest for optimizing regularization parameters *K* and *L*.

Secondly, we discuss the parameters of the RF. Based on previous studies, we chose PCA as Rotation Forest conversion method. Additionally, the J48 decision tree was selected as the base classifier from the WEKA database. In this experiment, two parameters (the number of feature subsets *K* and the number of decision trees *L*) were tested by the grid search method in the range of values to achieve better performance.  Figure 3 shows the prediction results of different parameters. We can see that accuracy fluctuates at the beginning and then is slowly enhanced with the increase of *L*, but it seems to be not closely related to the increase of *K*. Considering the accuracy rate and the time cost of the algorithm, as a result, we obtained optimal parameters of *K* = 20 and *L* = 3. For the* H. pylori* dataset, we use the AC to extract features and RF validation with the same parameters with the* Yeast* dataset.

**Figure 4:**
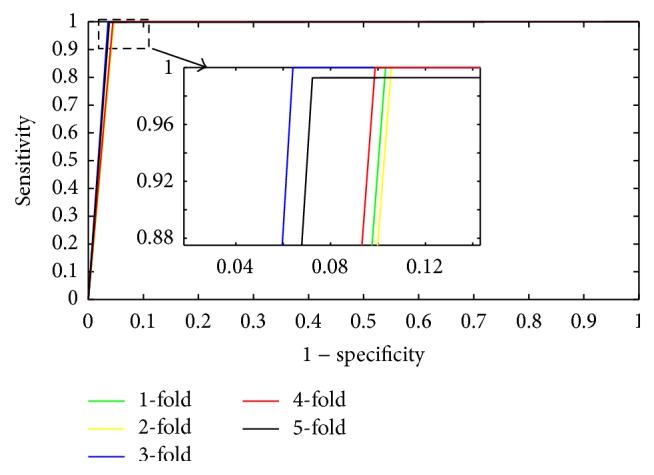
ROC curves performed by the proposed method on* Yeast* PPIs dataset.

The 5-fold cross-validation method was introduced to reduce the dependence of the data on the prediction model [51–55].  Table 1 lists all of the prediction results; the prediction accuracies were greater than 97.54%, the precisions were greater than 99.82%, and the sensitivities were greater than 95.01%. Our proposed method can yield an average prediction accuracy of 97.77 ± 0.29%. The ROC curves performed on* Yeast* dataset were shown in Figure 4. In this figure, *x*-ray depicts False Positive Rate (FPR) while *y*-ray depicts True Positive Rate (TPR).

### 3.3. Comparison with the Proposed Method on* H*.* pylori* Dataset

**Figure 5:**
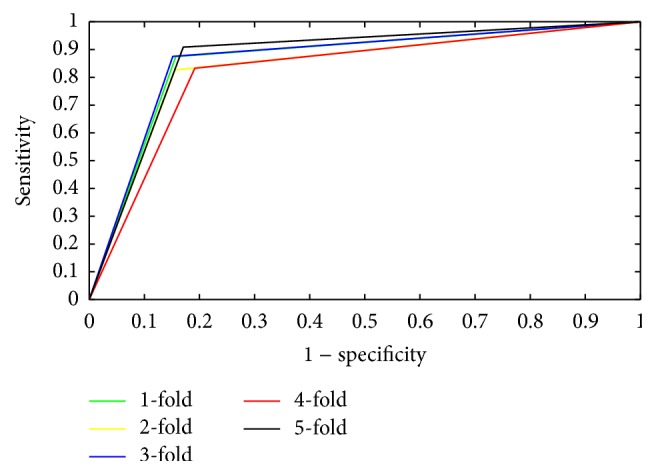
ROC curves performed by the proposed method on* H*.* pylori* dataset.

For analyzing the ability of the proposed method to predict PPIs, we tested its ability in different dataset. We used the proposed method to predict interactions on the* H*.* pylori* dataset. A total of 2916 proteins were included in this database, half of which were interacting pairs and the other half were noninteracting pairs. Our prediction results were shown in Table 2. We can see an accuracy, precision, sensitivity, and MCC of 84.84%, 86.36%, 82.77%, and 74.30%, respectively. The ROC curves performed on* H*.* pylori* dataset were shown in Figure 5.

### 3.4. Comparison with Previous Method

In order to more clearly assess the proposed method, we compared its results with the previous models on the* Yeast* dataset. As a classic classification algorithm, Support Vector Machine has a very superior performance in identifying interacting and noninteracting protein pairs. For example, Guo et al. [9] proposed a new method with Support Vector Machine combined with Autocovariance to predict Protein-Protein Interactions in* Yeast* dataset, and the results have proven its ability. Specifically, we use the same feature extraction method (AC) combined with PSSMs to compare the classification performance between Rotation Forest and SVM in the same dataset. We use grid search method to optimize the parameters of Support Vector Machine and set *c* = 0.5 and *g* = 0.6, respectively. The LIBSVM tools we adopted can be downloaded from https://www.csie.ntu.edu.tw/~cjlin/libsvm/. As can be seen from Table 3, when using SVM to predict PPIs of* Yeast* dataset, we obtained excellent results with the accuracy, precision, sensitivity, and MCC of 95.86%, 96.46%, 95.21%, and 92.06%, respectively. Most of the SVM based methods produce average standard values that were lower than our method on* Yeast* dataset.

In addition, we also compared the other existing methods on the* Yeast* and* H*.* pylori* datasets. Table 3 shows the average results of the other six methods in the* Yeast* dataset; we can see that the accuracy results obtained by these methods are between 75.08% and 89.33%. The average accuracy, precision, sensitivity, and MCC values of these methods are lower than those of our method, which are 97.77%, 99.96%, 95.57%, and 95.64%, respectively.  Table 4 shows the average predictive values of the six kinds of methods on the* H*.* pylori* dataset. We can see that the accuracy values obtained by these methods are between 75.80% and 87.50%, and the accuracy value of our proposed method is 84.84%, which also performs well in it.

### 3.5. Performance on Independent Dataset

Having achieved reasonably good results on the* Yeast* dataset and the* H. pylori* dataset, we decided to test the proposed method's performance on* independent datasets*. We built our final prediction model using all 11188 pairs of* Yeast* dataset as the training set with the parameters obtained by the grid search method; the value of *lg* is 40 in AC, the value of *K* is 20, and *L* is 3 in RF. The feature vector uses the feature extraction method (AC) based on the PSSMs to extract from the four datasets as RF test input. Independent test dataset is composed of the four databases (*C*.* elegans*,* E*.* coli*,* H*.* sapiens*, and* M*.* musculus*) collected in DIP database. The results of our model are listed in Table 5; the prediction accuracies on* C*.* elegans*,* E*.* coli*,* H*.* sapiens*, and* M*.* musculus* are 96.01%, 97.73%, 98.30%, and 96.81%, respectively. Those results show the excellent performance of our approach in predicting the accuracy of the interactions of other species.

## 4. Conclusions

In this study, a stable and robust computational method based on the features extracted from PSSM has been proposed to predict PPIs. It is known that the main computational challenge for sequence-based methods for predicting PPIs is to find a suitable feature representation to fully describe the important information of protein interactions. To solve this problem, we here firstly extracted the features from the Position-Specific Scoring Matrices (PSSMs) using Autocovariance (AC) method. Then, Rotation Forest (RF) model is employed as a novel and accurate classifier for PPIs prediction with better performance than state-of-the-art SVM classifier. In order to evaluate the performance of the proposed method, five PPIs datasets, that is,* C*.* elegans*,* E*.* coli*,* H*.* pylori*,* H*.* sapiens*, and* M*.* musculus*, have been used to perform the comparisons. As expected, the experiments results showed that the proposed method performs better than the other methods. Consequently, the proposed approach can be considered as a powerful tool for predicting PPI.

## Figures and Tables

**Table 1:** 5-fold cross-validation results obtained by using the proposed method on *Yeast *dataset.

Testing set	Accu. (%)	Prec. (%)	Sen. (%)	MCC (%)
1	97.59	100.00	95.14	95.28
2	97.54	100.00	95.03	95.19
3	98.17	100.00	96.40	96.40
4	97.59	100.00	95.01	95.27
5	97.99	99.82	96.27	96.06

*Average*	*97.77 ± 0.29*	*99.96 ± 0.08*	*95.57 ± 0.70*	*95.64 ± 0.55*

**Table 2:** 5-fold cross-validation results obtained by using the proposed method on *H. pylori *dataset.

Testing set	Accu. (%)	Prec. (%)	Sen. (%)	MCC (%)
1	85.76	87.45	82.87	75.52
2	83.53	82.65	84.38	72.49
3	86.11	87.55	83.57	76.02
4	81.99	83.27	79.51	70.42
5	86.82	90.88	83.55	77.06

*Average*	*84.84 ± 2.01*	*86.36 ± 3.40 *	*82.77 ± 1.90*	*74.30 ± 2.76*

**Table 3:** Different methods on *Yeast *dataset performance comparison.

Model	Test set	Accu. (%)	Prec. (%)	Sen. (%)	MCC (%)
Guo et al.'s work [9]	ACC	89.33 ± 2.67	88.87 ± 6.16	89.93 ± 3.68	N/A
	AC	87.36 ± 1.38	87.82 ± 4.33	87.30 ± 4.68	N/A

You et al.'s work [25]	PCA-EELM	87.00 ± 0.29	87.59 ± 0.32	86.15 ± 0.43	77.36 ± 0.44

Yang et al.'s work [56]	Cod1	75.08 ± 1.13	74.75 ± 1.23	75.81 ± 1.20	N/A
	Cod2	80.04 ± 1.06	82.17 ± 1.35	76.77 ± 0.69	N/A
	Cod3	80.41 ± 0.47	81.86 ± 0.99	78.14 ± 0.90	N/A
	Cod4	86.15 ± 1.17	90.24 ± 0.45	81.03 ± 1.74	N/A

Zhou et al.'s work [57]	SVM + LD	88.56 ± 0.33	89.50 ± 0.60	87.37 ± 0.22	77.15 ± 0.68

*Our method*	*SVM + PSSM*	*95.86 ± 0.34*	*96.46 ± 0.50*	*95.21 ± 0.70*	*92.06 ± 0.62*
	*RF + PSSM*	*97.77 ± 0.29*	*99.96 ± 0.08*	*95.57 ± 0.70*	*95.64 ± 0.55*

**Table 4:** Different methods on *H. pylori *dataset performance comparison.

Model	Accu. (%)	Prec. (%)	Sen. (%)	MCC (%)
Phylogenetic bootstrap [35]	75.80	80.20	69.80	N/A
HKNN [36]	84.00	84.00	86.00	N/A
Ensemble of HKNN [37]	86.60	85.00	86.70	N/A
Signature products [26]	83.40	85.70	79.90	N/A
Boosting [38]	79.52	81.69	80.37	70.64
Ensemble ELM [25]	87.50	86.15	88.95	78.13
*Our method*	*84.84 *	*86.36 *	*82.77 *	*74.30 *

**Table 5:** Prediction results in *independent datasets*.

Species	Test pairs	Accu. (%)
*C. elegans*	4013	96.01
*E. coli*	6954	97.73
*H. sapiens*	1412	98.30
*M. musculus*	313	96.81
